# Supplementary material for: Mobilome and Resistome Reconstruction from Genomes Belonging to Members of the Bifidobacterium Genus
Source: Microorganisms. 2019 Dec 2;7(12):638. doi: 10.3390/microorganisms7120638 (PMC6956390; doi:10.3390/microorganisms7120638)
Supplement: Supplementary file 1 [file microorganisms-07-00638-s001.zip › Figure_S1.docx]

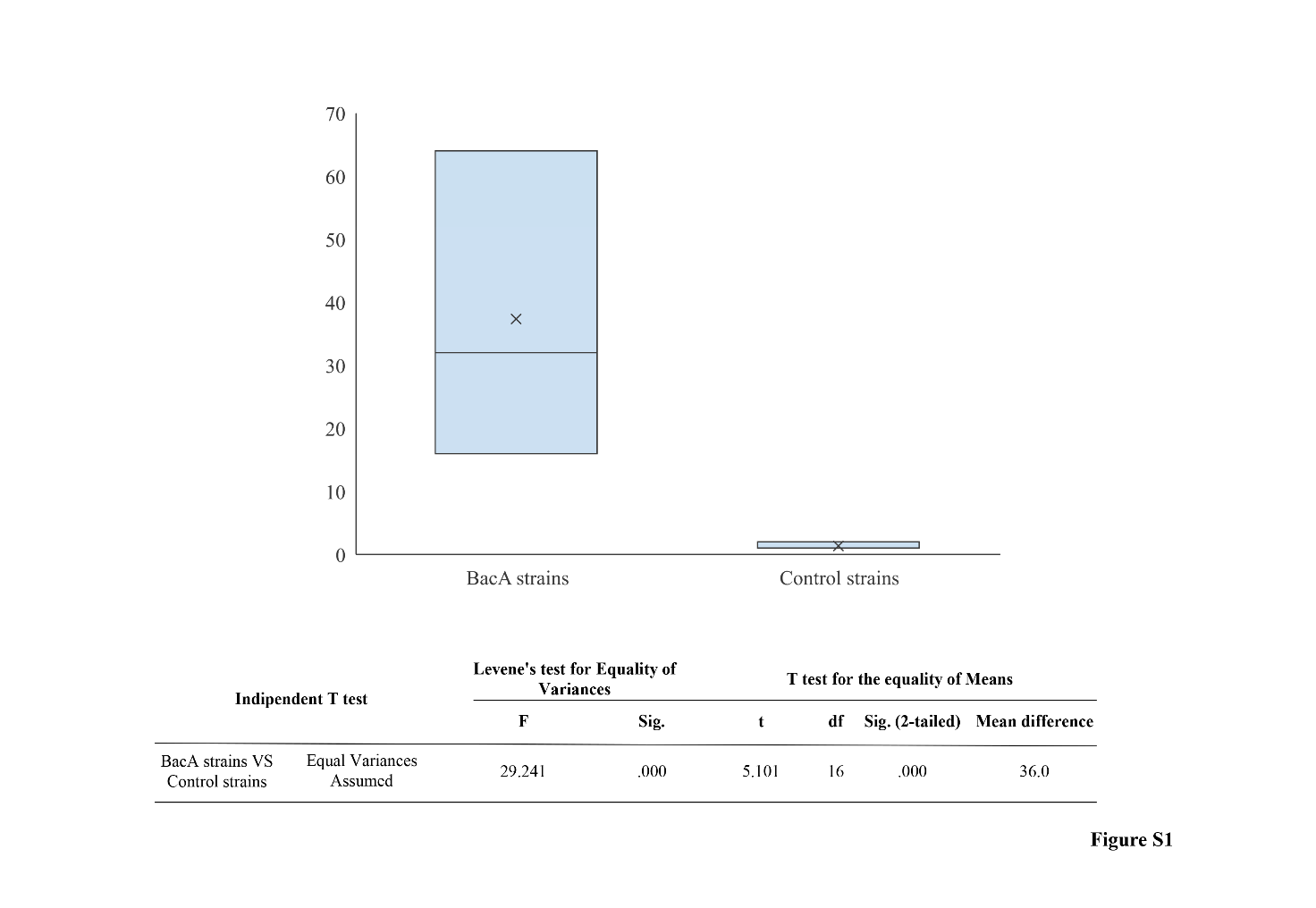
**Figure S1. Bacitracin A antibiotic breakpoint values.** Whiskers plot based on Bacitracin A antibiotic MIC breakpoint values of BacA strains group and Control group with a P value <0.001 (Student *t* test). The *x* axis represents the MIC values (microgram per milliliter), while the *y* axis shows the two groups analyzed- the boxes represent 50% of the data set, distributed between the first and the third quartiles. The median divides the boxes into the interquartile range, while the “X” represents the mean. The Student *t* test values are reported.
